# The effect of the visceral fat area on the predictive accuracy of C‐reactive protein for infectious complications after laparoscopy‐assisted gastrectomy

**DOI:** 10.1002/ags3.12329

**Published:** 2020-03-31

**Authors:** Michihisa Iida, Shigeru Takeda, Yuki Nakagami, Shinsuke Kanekiyo, Chiyo Nakashima, Mitsuo Nishiyama, Shin Yoshida, Nobuaki Suzuki, Shigefumi Yoshino, Hiroaki Nagano

**Affiliations:** ^1^ Department of Gastroenterological, Breast and Endocrine Surgery Yamaguchi University Graduate School of Medicine Yamaguchi Japan; ^2^ Oncology Center Yamaguchi University Hospital Yamaguchi Japan

**Keywords:** C‐reactive protein, gastrectomy, intra‐abdominal fat, postoperative complications, stomach neoplasms

## Abstract

**Aim:**

To investigate the influence of visceral fat area on postoperative C‐reactive protein levels and whether it affects its ability to diagnose infectious complications after laparoscopy‐assisted gastrectomy.

**Methods:**

A total of 435 consecutive patients who underwent laparoscopy‐assisted resection for gastric cancer from 2008 to 2017 were reviewed and divided into four groups according to visceral fat area quartiles. We evaluated the relationship between C‐reactive protein and visceral fat area and whether visceral fat area affects the sensitivity and specificity of C‐reactive protein in diagnosing postoperative infectious complications.

**Results:**

Postoperative C‐reactive protein levels increased with increasing visceral fat areas at every postoperative assessment. Multiple linear regression revealed that levels on postoperative day 3 significantly positively correlated with visceral fat area. Postoperative day 3 levels also showed moderate accuracy for diagnosing infectious complications (area under the curve, 0.78; sensitivity, 0.86; specificity, 0.65), with an optimal cut‐off of 11.8 mg/dL. The sensitivity for predicting infectious complications was low in the 1st visceral fat area quartile group but high in the 2nd, 3rd, and 4th groups (0.43 vs 1.0 vs 1.0 vs 0.94, respectively). By contrast, the specificity was high in the 1st and 2nd group but low in the 3rd and 4th (0.84 vs 0.70 vs 0.54 vs 0.48, respectively).

**Conclusion:**

Visceral fat area positively correlated with postoperative C‐reactive protein levels and this affected its accuracy in diagnosing infectious complications. A uniform C‐reactive protein cut‐off may not provide accurate predictions in patients with more extreme visceral fat areas.

## Graphical abstract

Visceral fat area was positively associated with postoperative C‐reactive protein levels and affected its accuracy in diagnosing infectious complications. A uniform C‐reactive protein cut‐off may not provide accurate predictions in patients with more extreme visceral fat areas.

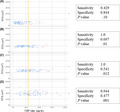

## 1 INTRODUCTION

Gastric cancer is the fourth most common malignancy and the second leading cause of cancer‐related death worldwide.^1^ Although surgery is the only curative treatment for gastric cancer, perioperative complications occur in 12.6%‐23.6% of cases.^2^, ^3^ Infectious complications occur in approximately 10% of patients after gastrectomy.^4^, ^5^ Recent studies have demonstrated that postoperative infectious complications (PICs) are associated with inferior long‐term survival in patients with various types of cancer, including gastric cancer.^5^, ^6^, ^7^ Artinyan et al^7^ reported that severe PICs are more closely associated with poor prognosis than less severe infectious complications. Therefore, it is important to detect PICs at an early stage before they progress, because timely diagnosis is key to improving long‐term survival after gastrectomy.

There are several approaches to predicting postoperative complications,^8^, ^9^, ^10^ including C‐reactive protein (CRP) levels after surgery.^11^, ^12^ Shishido et al^13^ reported that CRP on postoperative day (POD) 3 predicted infectious complications following gastric cancer resection. By contrast, a meta‐analysis found that CRP could not predict PICs after gastroesophageal cancer surgery.^14^ It is unclear whether prediction of PICs using CRP values is applicable for all patients.

Most recently, Okuma et al^15^ reported that postoperative CRP levels after minimally invasive esophagectomy positively correlated with visceral fat area (VFA). If postoperative CRP values after gastrectomy are affected by VFA, their ability to predict PICs may also be affected. Nevertheless, no studies have assessed whether VFA influences the predictive ability of CRP. Therefore, this retrospective analysis was conducted to determine the influence of VFA on postoperative CRP levels and whether it affects CRP’s ability to accurately diagnose PICs after laparoscopy‐assisted gastrectomy (LAG).

## 2 METHODS

### 2.1 Patients

The retrospective study included 435 consecutive patients who were pathologically diagnosed with primary gastric cancer and underwent LAG at Yamaguchi University Medical Hospital (Yamaguchi, Japan) between January 2008 and December 2017. Patients with other concomitant malignancies and those without preoperative computed tomography were excluded.

The patients were identified using our database and their detailed information was obtained from original medical records. This study was approved by the institutional review board of the Yamaguchi University Hospital (H28‐182).

### 2.2 Measurement of body composition parameters

We analyzed the subcutaneous fat area (SFA), VFA, and skeletal muscle area (SMA) on the preoperative multidetector computed tomography (MDCT) images using AZE Virtual Place Raijin software (Aze Ltd). VFA and SFA were measured using axial slices at the level of the umbilicus on preoperative multidetector computed tomography. Fat area was manually measured in the region with Hounsfield units (HU) within the range of −200 to −50. SMA was measured using axial slices at the level of the third lumbar vertebra. A threshold range of −30 to 150 HU was used to define muscle, and the SMA measurement included the abdominal, psoas, and paraspinal muscles. The skeletal muscle index (SMI) was calculated as SMA divided by height squared.

### 2.3 CRP measurement

Serum concentrations of CRP were measured on PODs 1, 3, 5, and 7. The quantitative determination of CRP was carried out using an automated analytical system (N‐Assay LA CRP‐T Nittobo, Nittoubo Medical Co., LTD). CRP <0.15 mg/dL was considered normal.

### 2.4 Definition of PICs

We defined PICs as either anastomotic leakage (extravasation of endoluminally administered water‐soluble contrast agent on radiography), abdominal abscess formation (intra‐abdominal pus collection confirmed by radiographic evidence or drainage), pancreatic fistula (drain output of any measurable volume of fluid on or after POD 3 with an amylase content greater than three times the serum amylase activity with medical management indicated [e.g. antibiotics/drainage]),^16^ incisional surgical site infection (infection of the superficial and deep incisional surgical site with medical management), or pneumonia (infection of the lungs diagnosed by radiographic evidence and sputum culture). According to the Clavien‐Dindo (CD) classification,^17^ the severity of PICs was classified as grades 0‐V, and patients with grade II or higher were defined as having PICs.

### 2.5 Surgical procedure

All patients underwent laparoscopic‐assisted distal gastrectomy or total gastrectomy (LATG) with D1, D1+, or D2 lymphadenectomy according to the Japanese Guidelines. After lymphadenectomy, a small‐laparotomy (<6 cm) was made in the upper abdomen for removal of the specimen and reconstruction. Omentectomy was performed in patients with sT3‐T4. The omentum more than 3 cm away from the gastroepiploic arcade and vessels of the omental branch was preserved in patients with sT1‐T2. Reconstruction after laparoscopic‐assisted distal gastrectomy was performed by the Billroth I, Billroth II, or Roux‐en Y approach, whereas Roux‐en Y reconstruction was always used after LATG. After reconstruction, a closed drain system was placed in subhepatic area.

### 2.6 Statistical analysis

All statistical analyses were performed using SPSS version 25.0 (SPSS Inc). Categorical variables are presented as number (percentage) and analyzed by the χ^2^ test or Fisher's exact test. All continuous variables are presented as means ± standard error and were analyzed using the Student's *t*‐test, Mann‐Whitney test, or Kruskal‐Wallis test. Multiple linear regression analysis was used to assess the effects of clinical factors on postoperative serum CRP levels. We included age, sex, Brinkman index, use of steroids, presence of liver damage, preoperative CRP, body mass index, SFA, VFA, SMI, pStage, type of resection, extent of node dissection, omentectomy, length of small laparotomy, operative duration, operative blood loss, incisional SSI, and PICs as confounders. Receiver operating characteristic analysis (ROC) was performed to determine the accuracy of CRP and WBC levels on POD1 and POD3 in diagnosing PICs by evaluating the area under the curve (AUC).^18^ The optimal cut‐off values were determined by maximizing Youden's index (defined as sensitivity + specificity − 1).^19^ *P* < .05 was considered statistically significant.

## 3 RESULTS

### 3.1 Patient characteristics

A total of 435 patients undergoing laparoscopic surgery for gastric cancer were identified. Twenty‐one patients were excluded for unavailability of preoperative computed tomography images (19 patients) or other concomitant cancer (two patients). Of the remaining 414 patients, 341 underwent laparoscopic‐assisted distal gastrectomy, and 73 patients underwent LATG. Patient characteristics are summarized in Table 1. PICs of grade II or greater according to the CD classification occurred in 35 patients (8.5%), including 15 with anastomotic leakage, 12 with abdominal abscess, two with pancreatic fistula, and six with pneumonia. Six cases (1.5%) developed superficial and deep incisional SSI, all of which were grade I according to CD classification. Onset of PICs were diagnosed between 3 and 12 days after gastrectomy (median, 6 days).

**Table 1:** Clinicopathological findings

Clinicopathological findings	Total (n = 414)	Visceral fat area				*P* value
		First quartile (n = 103)	Second quartile (n = 103)	Third quartile (n = 104)	Fourth quartile (n = 104)	
Age	66.1 ± 12.1	63.0 ± 14.2	67.3 ± 12.3	66.5 ± 11.2	67.73 ± 9.5	.023*
Gender						
Male	283	54	60	76	93	.000*
Female	131	49	43	28	11	
Smoking history						
No	181	60	53	37	31	.000*
Yes	233	43	50	67	73	
Use of steroids						
No	402	101	100	99	102	.565
Yes	12	2	3	5	2	
Presence of liver damage						
No	398	102	97	101	98	.198
Yes	16	1	6	3	6	
BMI (kg/m^2^)	22.7 ± 3.1	19.9 ± 2.1	22.0 ± 2.1	23.6 ± 2.6	25.4 ± 3.1	.000*
SFA (cm^2^)	121.2 ± 104.0	68.5 ± 46.3	112.7 ± 51.2	152.1 ± 176.4	150.8 ± 52.9	.000*
L3 SMI (cm^2^/m^2^)	44.7 ± 9.2	40.9 ± 8.1	42.9 ± 7.4	46.0 ± 9.8	49.1 ± 9.2	.000*
pStage						
I	367	92	89	97	89	.209
II	30	7	10	3	10	
III	15	4	4	2	5	
IV	2	0	0	2	0	
Type of resection						
Subtotal gastrectomy	341	88	86	88	79	.255
Total gastrectomy	73	15	17	16	25	
Type of reconstruction						
Billloth I	277	79	66	70	62	.045*
Billroth II	21	2	3	9	7	
Roux‐en Y	116	22	34	25	35	
Extent of node dissection						
D1·D1+	340	83	86	88	83	.773
D2	74	20	17	16	21	
Omentectomy						
No	385	97	98	99	91	.154
Yes	28	6	5	5	12	
Size of small‐laparotomy (mm)	53.8 ± 8.2	51.5 ± 7.7	52.8 ± 9.2	54.5 ± 7.4	56.5 ± 7.9	.000*
Operative duration (min)	336.1 ± 75.3	304.7 ± 56.3	317.1 ± 65.4	342.1 ± 71.2	380.2 ± 83.3	.000*
Operative blood loss (mL)	189.7 ± 236.1	122.4 ± 140.7	164.6 ± 178.8	175.1 ± 238.1	295.9 ± 314.2	.000*
Incisional surgical site infection						
No	408	100	103	103	102	.335
Yes	6	3	0	1	2	
Postoperative complication (>CD Grade I)						
No	344	84	89	93	78	.032*
Yes	70	19	14	11	26	
Postoperative infectious complication (>CD Grade I)						
No	379	96	99	98	86	.002*
Yes	35	7	4	6	18	
Hospital stay	17.7 ± 10.8	18.5 ± 13.2	16.9 ± 9.0	15.8 ± 5.1	19.8 ± 13.3	.040*

Note

Data are presented as mean ± SD or number.

Abbreviations: BMI, body mass index; CD, Clavien‐Dindo classification; SD, standard deviation; SFA, subcutaneous fat area; SMA, skeletal muscle area.

* Statistical significance (*P* < .05).

### 3.2 Clinicopathological findings stratified by VFA quartile

Patients were divided into four groups according to their VFA quartile (Table 1). The proportion of women decreased as the VFA increased. Higher VFA was correlated with longer operation time and increased operative blood loss. The incidence of postoperative complications in the 4th VFA quartile of group was significantly higher than in those of the other groups.

### 3.3 Postoperative serum CRP levels

**Figure 1:**
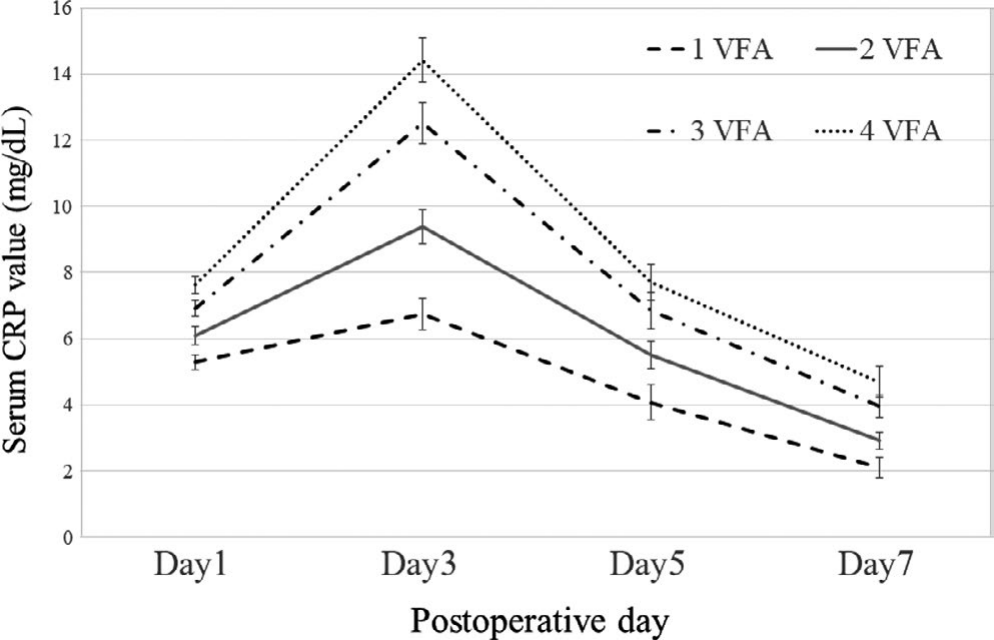
Postoperative changes in mean serum C‐reactive protein (CRP) levels (mg/dL) in each quartile group of visceral fat area (VFA, cm^2^). The error bars indicate the standard errors. 1 VFA, first quartile of VFA; 2 VFA, second quartile of VFA; 3 VFA, third quartile of VFA; 4 VFA, fourth quartile of VFA

Figure 1 shows the temporal change in postoperative CRP level for each VFA group. Serum CRP levels peaked 3 days after surgery, and then gradually decreased in all VFA groups. CRP levels increased with increasing VFA at every postoperative assessment (POD1, 5.3 vs 6.1 vs 7.0 vs 7.6 mg/dL, *P *= .00; POD3, 6.7 vs 9.4 vs 12.5 vs 14.4 mg/dL, *P *= .00; POD5, 4.0 vs 5.5 vs 6.9 vs 7.8 mg/dL, *P *= .00; and POD7, 2.1 vs 2.9 vs 4.0 vs 4.8 mg/dL, *P *= .00, respectively).

### 3.4 Presence/absence of PICs and postoperative serum CRP level in each VFA group

**Figure 2:**
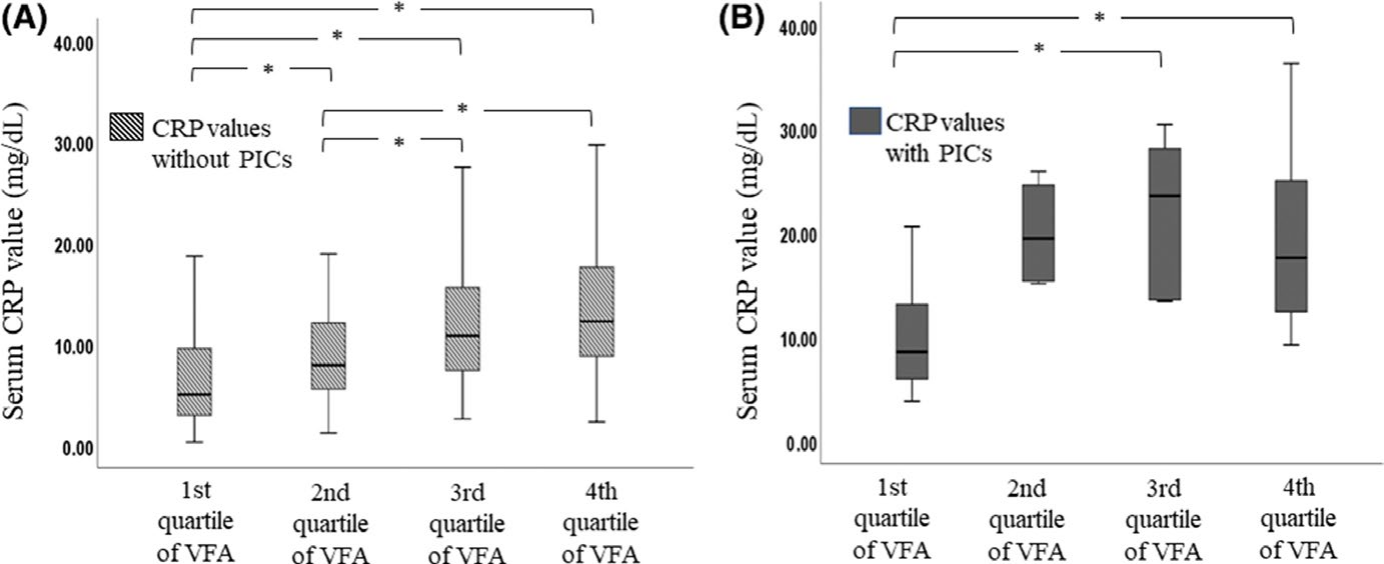
A, Serum C‐reactive protein (CRP) levels (mg/dL) on postoperative day 3 (POD3) in patients without postoperative infectious complications (PICs) in each quartile group of visceral fat area (VFA, cm^2^). B, Serum CRP levels on POD3 in patients with PICs in each quartile group of VFA. Mild outlier, interquartile range (IQR) 1.5‐3.0 IQR; *Extreme outlier (IQR> 3.0 IQR); *P < .05

Figure 2 shows the box‐and‐whisker plot of CRP levels on POD3 for each VFA group according to the incidence of PICs. The median CRP values on POD3 for each VFA group according to the presence of PICs are shown in the Table S1. The CRP level on POD3 increased in a stepwise manner with VFA increases in patients with and without PICs. In patients without PICs, the median CRP level within the fourth VFA quartile was significantly higher than that in the first and second, as were median levels in the third quartile.

### 3.5 Effect of VFA on CRP on POD3 according to simple and multiple regression analysis

We examined the effect of factors that had been reported to be useful for predicting infectious complications after gastric cancer surgery on CRP levels on POD3 (Table 2). On simple linear regression analysis, sex, Brinkman index, BMI, SFA, VFA, SMI, type of resection, omentectomy, length of small‐laparotomy, operative time, operative duration, and PICs were significantly correlated with CRP on POD3, but not with age, use of steroids, presence of liver damage, preoperative CRP, stage, extent of node dissection, or incisional SSI. On simple linear regression analysis, VFA showed the highest correlation coefficient with CRP on POD3 among the clinicopathological factors analyzed. Multiple linear regression analysis demonstrated that sex, VFA, type of resection, and PICs were independently associated with the postoperative CRP on POD3.

**Table 2:** Simple and multiple linear regression analysis of factors affecting postoperative CRP

Variables (reference)	Simple linear regression analysis				Multiple linear regression analysis		
	β	95% Confidence interval	*R* ^2^	*P* value	Regression coefficient	95% Confidence interval	*P* value
Age	0.061	−0.68 to 0.291	.004	.222			
Sex (male vs female)	0.327	−0.030 to −0.017	.107	.000*	−1.799	−3.923 to −0.818	.003*
Brinkman index	0.184	0.001 to 0.003	.034	.000*	0.000	−0.001 to 0.001	.615
Use of steroids (absent vs present)	0.091	−7.315 to 0.225	.008	.065			
Presence of liver damage (absent vs present)	0.077	−5.916 to 0.654	.003	.116			
Preoperative CRP	0.048	−0.798 to 1.857	.002	.433			
BMI	0.352	0.124 to 0.209	.124	.000*	0.199	−0.084 to 0.481	.168
SFA	0.119	0.358 to 3.403	.014	.016*	0.001	−0.007 to 0.005	.797
VFA	0.423	4.050 to 6.588	.177	.000*	0.025	0.012 to 0.039	.000*
SMI	0.283	0.265 to 0.524	.08	.000*	0.007	−0.070 to 0.084	.867
pStage (I vs II, III, IV)	0.081	−0.001 to 0.009	.004	.101			
Type of resection (subtotal vs total)	0.241	0.009 to 0.019	.058	.000*	1.896	0.034 to 3.504	.034*
Extent of node dissection (D1, D1+ vs D2)	0.074	−0.001 to 0.010	.006	.132			
Omentectomy (absent vs present)	0.133	0.951 to 5.971	.017	.007*	1.234	−1.037 to 3.504	.286
Length of small‐laparotomy	0.272	0.141 to 0.289	.074	.000*	0.047	−0.027 to 0.122	.214
Operative duration	0.381	3.340 to 5.402	.143	.000*	0.008	−0.003 to 0.018	.150
Operative blood loss	0.221	4.545 to 11.337	.049	.000*	0	−0.004 to 0.002	.490
Incisional SSI	0.015	−4.496 to 6.131	.000	.762			
Postoperative infectious complication (absent vs present)	0.325	5.507 to 9.825	.106	.000*	4.271	2.449 to 6.627	.000*

Abbreviations: BMI, body mass index; CRP, C‐reactive protein; SFA, subcutaneous fat area; SMA, skeletal muscle area; SSI, surgical site infection.

* Statistical significance (*P* < .05).

### 3.6 Diagnostic accuracy of CRP and WBC on POD1 and POD3 for the PICs prediction

**Figure 3:**
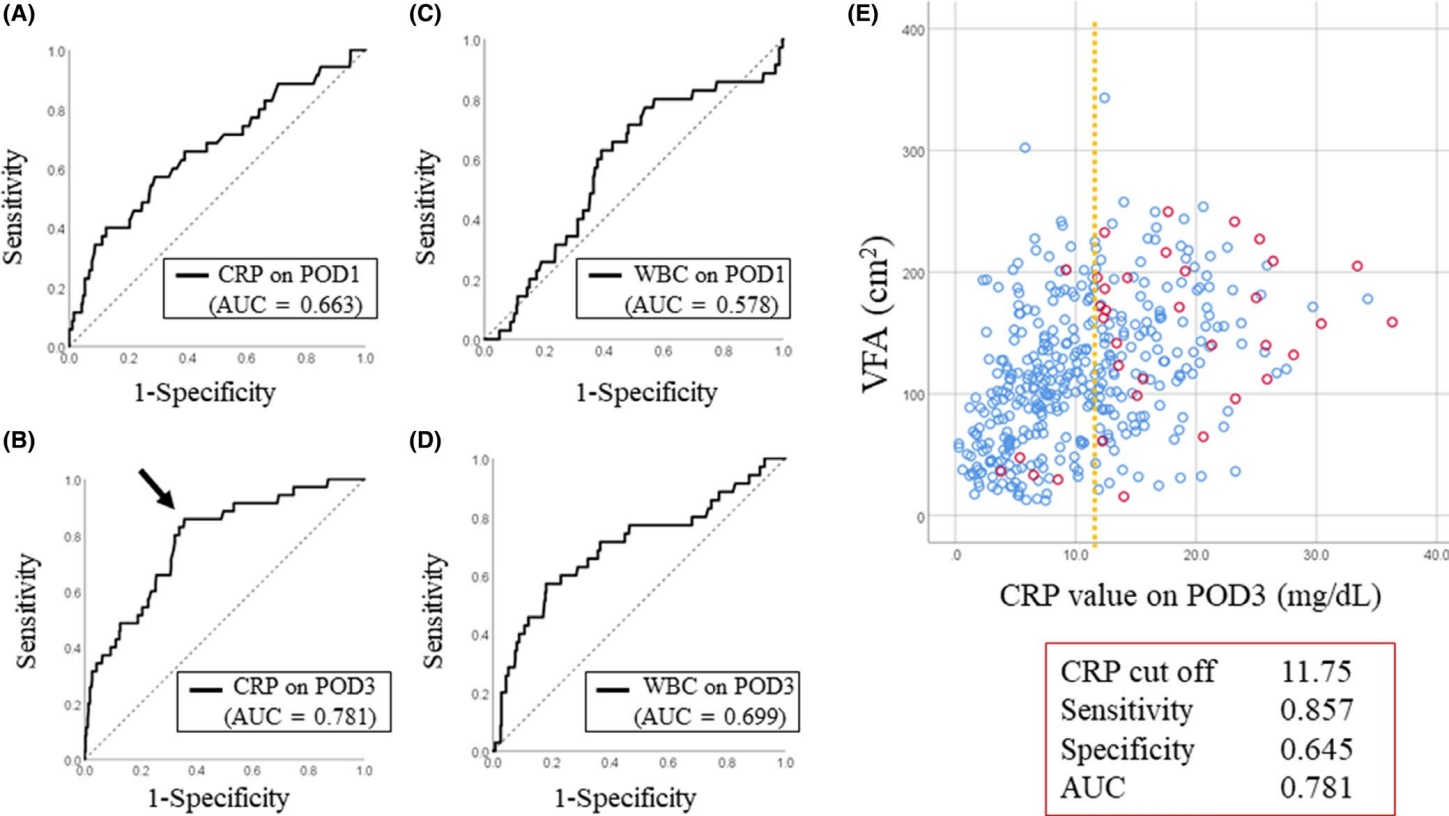
A‐D, Receiver operating characteristic (ROC) curve showing the optimal cut‐off level (mg/dL) of (A) C‐reactive protein (CRP) on postoperative day 1 (POD1), (B) CRP on POD3, (C) WBC on POD1, (D) WBC on POD3 for the prediction of postoperative infectious complications (PICs). The CRP level on POD3 had the best diagnostic accuracy and revealed moderate diagnostic accuracy (area under the ROC curve, 0.781; sensitivity, 85.7; specificity, 64.5). The arrow indicates the optimal cut‐off point. E, Scatter plots of visceral fat area (VFA, cm^2^) and CRP levels on POD3. The blue and red colors indicate patients without PICs and with PICs, respectively. Dotted lines indicate the cut‐off value of CRP on POD3

To determine the most useful biochemical tests of the acute systemic inflammatory response to diagnose PICs, we performed the ROC analysis of PICs prediction using CRP and WBC value on POD1 and POD3. The area under the ROC curve (AUC) of CRP on POD1, CRP on POD3, WBC on POD1, and WBC on POD3 were as follows: 0.663, 0.781, 0.578, and 0.699, respectively (Figure 3A‐D). Based on the AUC, CRP on POD3 had the best diagnostic accuracy and revealedmoderately accurate (AUC, 0.781; 95% confidence interval, 0.704‐0.859; sensitivity, 85.7%; specificity, 64.5%), with a calculated optimal cut‐off value of 11.8 mg/dL (Figure 3B). Figure 3E shows the scatter plots of VFA and CRP levels on POD3 according to PIC status.

### 3.7 Univariate and multivariate analysis for factors related to PICs

We examined clinicopathological factors related to the development of PIC after gastrectomy. Table 3 shows the results of univariate and multivariate analyses of clinicopathological factor for PICs prediction. On univariate analysis, gender (*P *= .007), smoking history (*P *= .009), VFA (*P *= .003), SMI (*P *= .014), type of resection (*P *= .000), omentectomy (*P *= .023), length of small‐ laparotomy (*P *= .007), operative duration (*P *= .000), operative blood loss (*P *= .013), and CRP on POD 3 of 11.75 mg/dL or greater (*P *= .000) were significantly associated with the development of PICs after LAG. Multivariate analysis revealed that operative duration and CRP on POD 3 of 11.75 mg/dL or greater were independent risk factors for development of PICs, with odds ratios (95% CI) of 1.007 (1.000‐1.014) and 6.333 (2.254‐17.92).

**Table 3:** Univariate and multivariate analysis of factors associated with postoperative infectious complications

Clinicopathological findings	Univariate analysis			Multivariate analysis		
	Postoperative infectious complications (CD grade >I)			Odds ratio	95% CI	*P* value
	Negative (n = 379)	Positive (n = 35)	*P* value			
Age	66.2 ± 12.2	67.8 ± 10.1	.449			
Gender						
Male	252	31	.007*	1.145	0.280‐4.676	.851
Female	127	4		1		
Smoking history						
No	173	8	.009*	1		.692
Yes	206	27		1.23	0.440‐3.438	
Use of steroids						
No	368	34	.731			
Yes	11	1				
Presence of liver damage						
No	365	33	.553			
Yes	14	2				
BMI (kg/m^2^)	22.6 ± 3.0	23.8 ± 3.8	.083			
SFA (cm^2^)	120.0 ± 106.6	134.0 ± 68.3	.445			
VFA (cm^2^)	114.6 ± 58.4	146.1 ± 66.2	.003*	1.001	0.993‐1.008	.868
L3 SMI (cm^2^/m^2^)	44.4 ± 9.2	48.4 ± 7.7	.014*	1.002	0.957‐1.049	.938
Type of resection						
Subtotal gastrectomy	320	21	.000*	1		.948
Total gastrectomy	59	14		1.036	0.352‐3.049	
Extent of node dissection						
D1·D1+	313	27	.274			
D2	66	8				
Omentectomy						
No	357	29	.023*	1		.813
Yes	22	6		1.17	0.319‐4.287	
Size of small‐laparotomy (mm)	53.5 ± 8.1	57.5 ± 9.2	.007*	1.008	0.966‐1.051	.715
Operative duration (min)	330.3 ± 71.5	402.5 ± 86.1	.000*	1.007	1.000‐1.014	.045*
Operative blood loss (mL)	177.5 ± 140.7	322.3 ± 320.3	.013*	1	0.999‐1.002	.504
CRP on POD3						
<11.75 (mg/dL)	244	135	.000*	1		.000*
>11.75 (mg/dL)	5	30		6.333	2.254‐17.792	

Note

Data are presented as mean ± SD or number.

Abbreviations: BMI, body mass index; CD, Clavien‐Dindo classification; CRP, C‐reactive protein; POD, postoperative day; SD, standard deviation; SFA, subcutaneous fat area; SMA, skeletal muscle area.

* Statistical significance (*P* < .05).

### 3.8 Diagnostic accuracy of CRP stratified by VFA quartile

**Figure 4:**
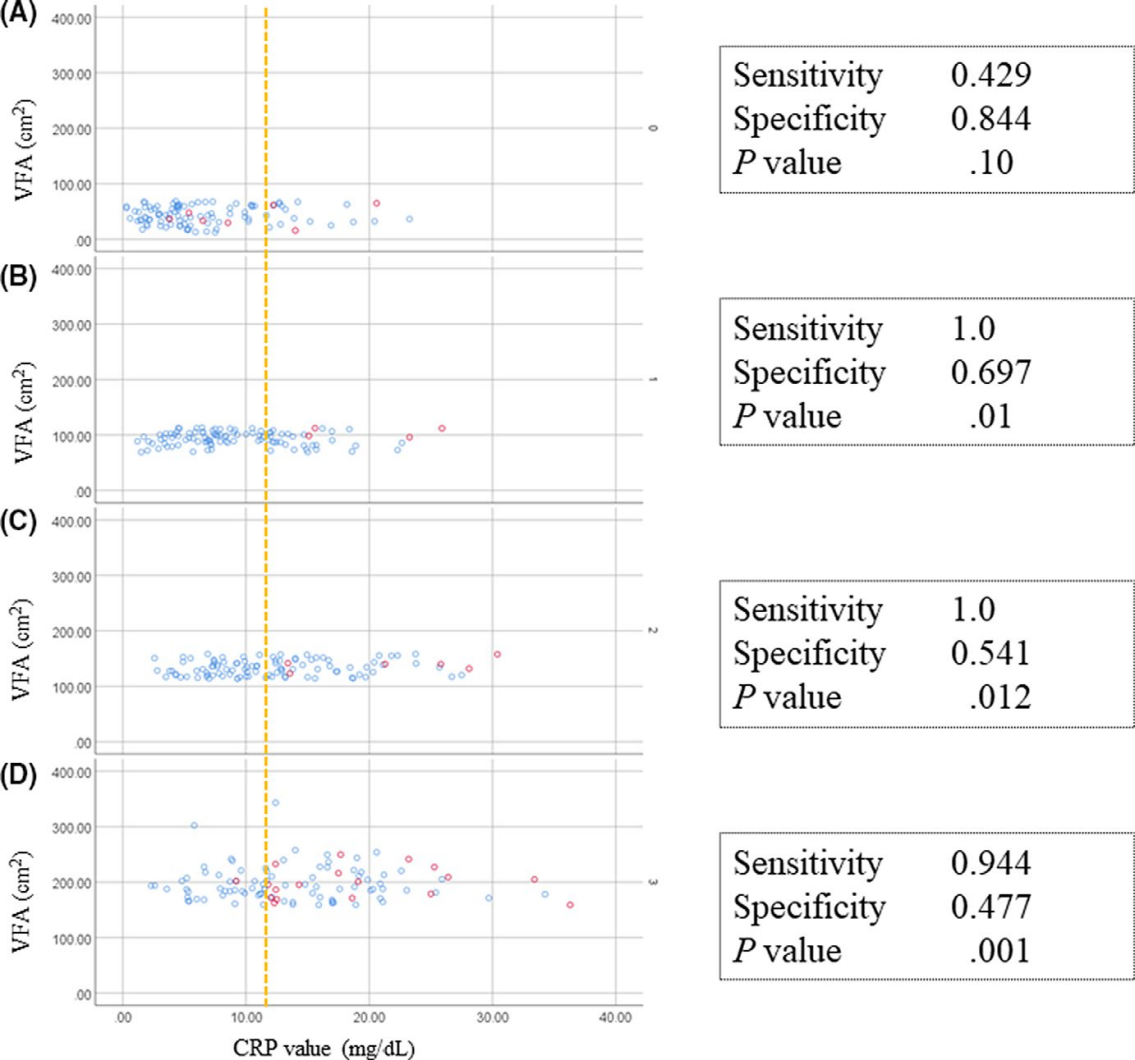
Scatter plots of visceral fat area (VFA, cm^2^) and C‐reactive protein (CRP) levels (mg/dL) on postoperative day 3 (POD3) in each quartile group of VFA. A, First quartile of VFA, B, second quartile of VFA, C, third quartile of VFA, D, forth quartile of VFA. The blue and red colors indicate patients without PICs and with PICs, respectively. Dotted lines indicate the cut‐off value of CRP on POD3

Figure 4 shows scatter plots of VFA and CRP levels on POD3 according to PIC status for each VFA quartile. CRP levels in the 1st quartile tended to be low, with four out of seven patients with PICs having lower CRP values than the cut‐off value. Conversely, CRP values in the 4th quartile tended to be high, with 35 out of 76 patients without PICs having higher CRP values than the cut‐off value. As shown in Table 4, the sensitivity for PICs in the 2nd, 3rd, and 4th quartile groups was high while it was low in the 1st quartile group (0.43 vs 1.0 vs 1.0 vs 0.94, respectively). In contrast, the specificity in the 1st and 2nd quartile groups was high while it was low in the 3rd and 4th quartile groups (0.84 vs 0.70 vs 0.54 vs 0.48, respectively). Figures S1 and S2 show scatter plots of VFA and CRP levels on POD3 according to PIC status for each VFA quartile of LADG and LATG, respectively. In the sub‐analysis limited to LADG (calculated optimal cut‐off value of 11.8 mg/dL), the specificity decreased as the quartile of VFA increased, and sensitivity was higher in groups other than the first quartile. Although the sub‐analysis, limited to LATG (calculated optimal cut‐off value of 12.0 mg/dL), had an inadequate number of cases and events, sensitivity was lowest in the first quartile and specificity was lowest in the fourth quartile.

**Table 4:** Accuracy of CRP levels on POD3 in diagnosing postoperative infectious complication

	n	Sensitivity	Specificity	PPV	NPV	*P*
All	414	0.857	0.645	0.182	0.98	.000*
First quartile of VFA	103	0.429	0.844	0.167	0.953	.100
Second quartile of VFA	103	1	0.697	0.118	1	.010*
Third quartile of VFA	104	1	0.541	0.118	1	.012*
Fourth quartile of VFA	104	0.944	0.477	0.274	0.976	.001*

Abbreviations: CRP, C‐reactive protein; NPV, negative predict value; POD, postoperative day; PPV, positive predict value; VFA, visceral fat area.

* Statistical significance (*P* < .05).

## 4 DISCUSSION

It is a known trend that the inflammatory response is high in obese patients, who show elevated serum levels of inflammatory cytokines, such as tumor necrosis factor‐α, interleukin‐6, and CRP.^20^, ^21^, ^22^ However, there are few reports about differences in the inflammatory response change after surgery between obese patients and non‐obese patients.

This study identified significant associations between VFA and postoperative CRP after LAG. Multiple linear regression analysis found that VFA, sex, type of resection, and PICs were factors affecting CRP levels on POD3, with VFA showing the strongest correlation. Previous studies reported that sex, surgical procedure, surgical approach, duration of the operation, amount of blood loss, and PICs were factors related to POD3 CRP levels after gastrectomy.^23^, ^24^ Although there have been no studies on the association between VFA and postoperative CRP levels after gastrectomy, a recent report revealed that VFA was significantly associated with increased serum CRP levels after minimally invasive esophagectomy.^15^ Previous reports have identified high VFA as a risk factor for the development of postoperative complications,^25^, ^26^ and, in this study, VFA was also identified as a risk factor for PICs by univariate analysis. This suggests a mechanism by which PICs are more likely to occur in patients with high VFA, resulting in higher CRP. However, in our study, a relationship between VFA and postoperative CRP was also observed in patients with or without PICs, suggesting that visceral obesity might promote the postoperative inflammatory response. Although the exact mechanism underlying visceral obesity's effect on the postoperative inflammatory responses is unknown, one study suggested that visceral fat itself is a source of inflammation. Fontana et al reported that plasma interleukin‐6 concentrations were significantly higher in the portal vein than in the peripheral blood of obese patients who underwent open gastric bypass surgery. They concluded that visceral fat may be an important source of postoperative interleukin‐6 production in obese people.^27^ Our finding, that postoperative CRP after gastrectomy increased with increasing VFA in patients, with or without postoperative complications, corroborates the findings of these studies. The CRP value on POD3 among patients in the 4th VFA quartile was more than twice that of the 1st VFA quartile, raising the question about the usefulness of CRP level to diagnose PICs in patients across the VFA spectrum.

Several studies demonstrated the moderate diagnostic accuracy of CRP for detecting PICs after gastrectomy. Although several studies have investigated the prediction of PICs by CRP after open and laparoscopic gastrectomy,^13^, ^14^, ^28^ few focused on laparoscopic gastrectomy only.^29^ As CRP levels on POD3 have been reported to be nearly twice as high after open gastrectomy than after laparoscopic gastrectomy,^24^ we only analyzed LAGs cases to ensure accurate results. Many previous studies demonstrated that CRP values on POD3^12^, ^13^, ^30^ or POD4^11^, ^14^, ^28^ were useful as cut‐off values for predicting PICs. In our cohort, the CRP value on POD3 showed the highest AUC among POD1, POD3, and POD5 (data not shown). In addition, PICs developed since POD3 in our cohort. Therefore, CRP value on POD3 was used as the cut‐off for PIC prediction.

We also demonstrated that the accuracy of diagnosing PICs by CRP was influenced by VFA. Patients in the second and third quartiles of VFA showed moderately high sensitivity and specificity. Conversely, the sensitivity in the lower VFA groups was worse than in the higher groups, while specificity in the higher groups was worse than in the lower groups. A similar trend was observed in the LADG and LATG subgroup analyses. Because postoperative serum CRP is elevated in patients with high VFA, with or without PICs, the specificity of PIC prediction is reduced when using a uniform CRP cut‐off value in patients with a high VFA. Similarly, because postoperative serum CRP levels are low in patients with low VFA, regardless of the presence of PICs, the prediction sensitivity is lower when a uniform cut‐off is used in patients with low VFAs. In other words, CRP value may not be an accurate predictor of PICs in patients with extreme VFAs when using a uniform cut‐off that is not affected by VFA. Although it is desirable to identify CRP cut‐offs that correspond to VFA values, this was not possible in our study because the sample size was small. When utilizing CRP levels for diagnosing PICs in daily clinical practice, it may be useful to recognize that false negatives are more common in patients with a low VFA while false positives are more common in patients with a high VFA. Understanding that CRP is affected by VFA may enable more sensitive PIC diagnosis. To the best of our knowledge, this is the first study to report that PIC prediction by CRP value is affected by VFA.

There are several potential limitations to this study. First, this was a retrospective study. Second, because the extent of resection was independently associated with CRP level in the multiple linear regression analysis, more rigorous studies examining total and partial gastrectomy separately are needed. In the subgroup analysis of this study, there were fewer cases and fewer events of LATG and we could not clearly show the relationship between the diagnostic accuracy of PICs and VFA. In addition, while defining the CRP cut‐off according to the VFA might improve the accuracy of PIC prediction, this was difficult to perform because of the limited number of infectious events in our cohort. Therefore, further studies with a larger number of patients should be conducted in the future.

In conclusion, the present study showed that VFA was positively associated with postoperative CRP levels and that this affected its accuracy in diagnosing infectious complications. Although understanding the limitations and optimal use of a simple biochemical marker such as CRP is crucial for improving outcomes, it may be possible to achieve a more accurate PICs diagnosis by considering VFA when using CRP as a predictive marker for PICs.

## DISCLOSURE

Conflict of Interest: Authors declare no conflict of interests for this article.

Ethical Approval: This study was approved by the institutional review board of the Yamaguchi University Hospital (H28‐182).

## Supporting information

Fig S1

Fig S2

Table S1
