# Supplementary figures and images for: The effect of the visceral fat area on the predictive accuracy of C‐reactive protein for infectious complications after laparoscopy‐assisted gastrectomy
Source: Ann Gastroenterol Surg. 2020 Mar 31;4(4):386–95. doi: 10.1002/ags3.12329 (PMC7382426; doi:10.1002/ags3.12329)

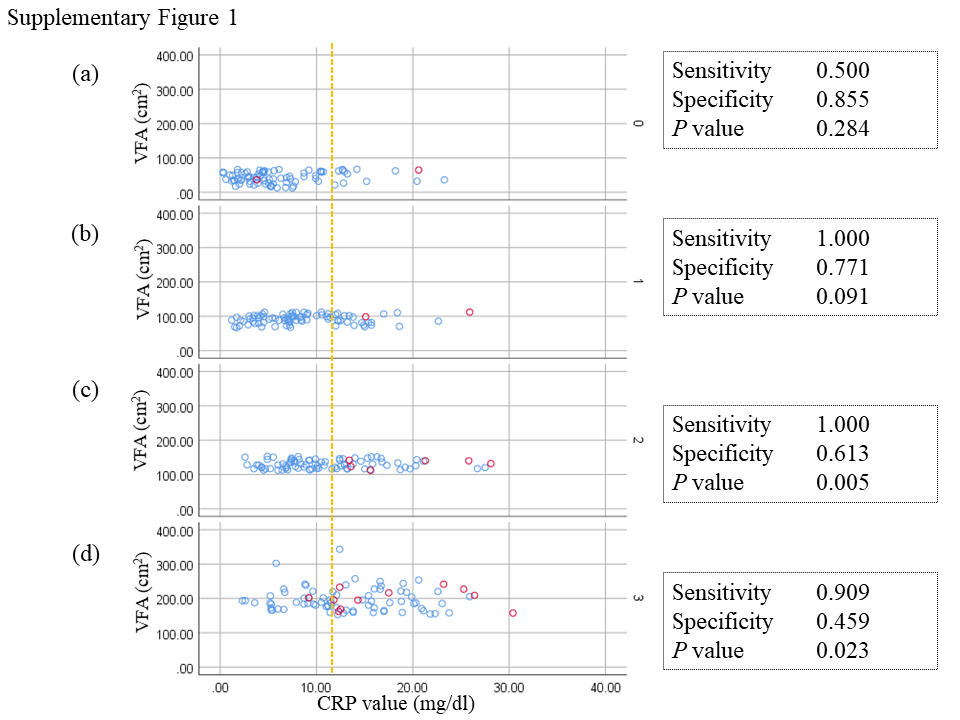

Supplement: Supplementary file 1 — Fig S1 [file AGS3-4-386-s001.tif]
